# Mapping Motor Neuroplasticity after Successful Surgical Brachial Plexus Reconstruction Using Navigated Transcranial Magnetic Stimulation (nTMS)

**DOI:** 10.3390/neurolint16010016

**Published:** 2024-02-01

**Authors:** Gregor Durner, Ina Ulrich, Alexandra Gerst, Ralf Becker, Christian Rainer Wirtz, Gregor Antoniadis, Maria Teresa Pedro, Andrej Pala

**Affiliations:** 1 Peripheral Nerve Surgery Unit, Department of Neurosurgery, University of Ulm, Lindenallee 2, 89312 Günzburg, Germanygregor.antoniadis@uni-ulm.de (G.A.);; 2 Department of Neurosurgery, University of Ulm, Lindenallee 2, 89312 Günzburg, Germanyrainer.wirtz@bkh-guenzburg.de (C.R.W.); 3 Department of Neuroradiology, University of Ulm, Lindenallee 2, 89312 Günzburg, Germany; ralf.becker@uni.ulm.de

**Keywords:** navigated transcranial magnetic stimulation, neuroplasticity, peripheral nerve surgery, brachial plexus injury, motor mapping

## Abstract

Brachial plexus reconstruction (BPR) consists of the complex surgical restoration of nerve structures. To further understand the underlying motor cortex changes and evaluate neuroplasticity after a successful surgery, we performed a navigated transcranial magnetic stimulation (nTMS) study mapping the postoperative motor representation of the formerly plegic arm. We conducted a prospective nTMS study mapping the musculocutaneous nerve as a representative, prominent target of BPR including a patient (*n* = 8) and a control group (*n* = 10). Measurements like resting motor threshold (RMT), cortical motor area location, and size were taken. Mathematical analysis was performed using MATLAB 2022, statistical analysis was performed using SPSS 26, and nTMS mapping was performed using the Nexstim NBS 5.1 system. Mapping was feasible in seven out of eight patients. Median RMT on the affected hemisphere was 41% compared to 50% on the unaffected hemisphere and they were 37% and 36% on the left and right hemispheres of the control group. The motor area location showed a relocation of bicep brachii representation at the middle precentral gyrus of the corresponding contralateral hemisphere. Motor area size was increased compared to the control group and the patient’s unaffected, ipsilateral hemisphere. Understanding cortical reorganization is important for potential future treatments like therapeutic nTMS. The issue of motor neuroplasticity in patients with brachial plexus lesions is worth exploring in further studies.

## 1. Introduction

The first characterizations of the motor cortex in humans using transcranial magnetic stimulation (TMS) date back to the 1990s and have provided us with a basic understanding of the TMS method and brain hodotopy [1,2,3]. Shortly after, examinations of motor neuroplasticity (MN) followed, reliably demonstrating changes in cortical motor representation in cases of blind patients reading Braille after upper limb amputation or ischemic nerve block [4,5,6]. More recently, reports on motor neuroplasticity after stroke [7,8,9] in patients with gliomas [10,11,12] and other pathologies have been added.

As a center specializing in the surgical restoration of the brachial plexus, MN changes are also relevant for us in the recovery process of our patients, which substantially differs from those of patients with other pathologies. In stroke or glioma removal, functional cortical motor tissue is permanently lost. The “aim” of MN here is to find a new location for motor function. In the case of amputation, the cortex is physically intact; however, the affected limb will never again be accessed by the formerly responsible cortical area. In the case of a successful brachial plexus surgery, however, a restoration of the thus-far-damaged motor pathway (from motor cortex to muscle) is achieved. Still, if the surgical intervention is performed after 3 to 6 months and if nerve growth of 1 mm/day can be assumed, signs of reinnervation can be expected 1 year after plexus trauma and deafferentation at the earliest. In this scenario, the formerly responsible cortical motor area is still intact.

Brachial plexus surgery offers a wide variety of reconstructive techniques [13,14,15]. Regarding nerve reconstruction, two different methods can be broadly differentiated: “anatomical reconstruction” and “nerve transfer”. In the case of anatomical reconstruction, the former nerve anatomy is restored, if necessary, with the use of a nerve graft. When performing nerve transfers, however, a donor nerve is sutured onto the proximally injured nerve—for example, the phrenic nerve onto the musculocutaneous nerve in the case of bicep palsy.

As the bicep muscle is one of the most important muscles in brachial plexus surgery, we chose it as our target for examining cortical motor representation and possible neuroplastic changes. To clarify which cortical motor areas are responsible for bicep activation in these cases, we conducted a pilot study using navigated transcranial magnetic stimulation (nTMS).

## 2. Materials and Methods

### 2.1. Study Design

The conducted examination was a prospective, single-center analysis. It was approved by our local ethics committee (Ulm University, nr. 472/19). After defining the bicep as our target muscle, we screened all brachial plexus surgeries (*n* = 384) performed at our neurosurgical department between 2010 and 2019 in order to identify patients who regained good bicep function, which was defined as reaching at least 3/5 on the Medical Research Council (MRC) scale for muscle strength. For the purpose of this study, nerve recovery needed to be completed; hence, we only chose patients with a finished postoperative routine follow-up, which we typically conduct up to 3 years after surgery. Eligible patients then received an invitation to take part in the examination. In order to find healthy volunteers for a control group, we invited participants with a notice at our university. The group size was initially set to 10 people for two groups: brachial plexus patients and healthy volunteers. Because three-dimensional (3D), non-contrast magnetic resonance imaging (MRI) served as a basis for TMS navigation, exclusion criteria included typical MRI contraindications like metal implants (cochlear implants, pacemakers, large tattoos, aneurysm clips, etc.) or pregnancy. Furthermore, nTMS-specific exclusion criteria were defined as known epilepsy or psychiatric disorders.

### 2.2. nTMS Protocol

For the 3D MRI sequence, a magnetization-prepared rapid gradient-echo (MPRAGE) sequence was obtained from each subject. nTMS mapping was then performed using the Nexstim NBS 5.1 system (Helsinki, Finland). Surface electromyography (EMG) electrodes (NeuroTab, spes medica, Genova, Italy) were used to continuously record bicep muscle responses. The system-integrated Nexstim EMG with an amplifier offers a frequency range from 10 to 500 Hz and a resolution of 0.3 μV. EMG responses were rated as positive if above 50 μV amplitude. EMG responses were automatically stored for later review if necessary.

The mapping process began by establishing a motor hotspot via rough mapping across the whole hemisphere. Consecutively, the subject’s resting motor threshold (RMT) was determined, and motor mapping of both hemispheres was performed in a standardized fashion with a maximum of 120% RMT [16]. In this process, the stimulation coil was moved circularly around the motor hotspot until no more motor-evoked potentials (MEPs) could be elicited, and the whole motor area was defined. The stimulation vector was set perpendicular to the gyrus border and otherwise in the dorsal–rostral direction.

Both hemispheres were mapped with running bicep EMGs on both arms (operated as well as on the healthy side) in order to detect possible neuroplastic ipsilateral innervation of the biceps. Positive motor responses were then entered into a cortical parcellation system, which was proposed by Corina et al. [17] to define the cortical localization of motor responses. Our hypothesis was that we would find reorganized motor areas at the precentral gyrus mostly.

### 2.3. Computation of the Cortical Representation Area and Statistical Analysis

**Figure 1:**
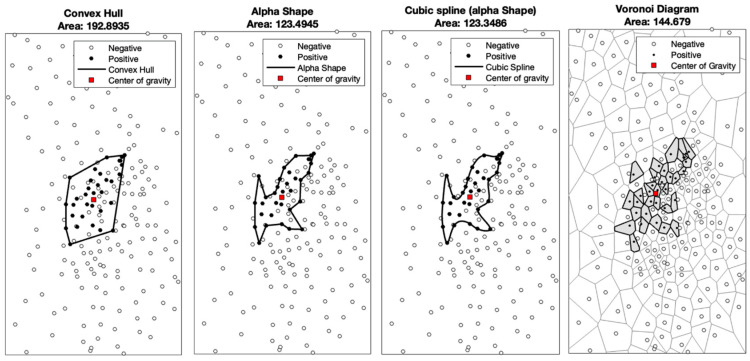
Illustrative cases of motor area size calculations with the convex hull, alpha shape, cubic spline (alpha shape), and Voronoi diagram methods.

In order to measure and compare the size of each motor area, 3D axis information of each stimulation spot was exported and later analyzed using MATLAB R2022 (The MathWorks Inc., Natick, MA, USA). As an area around a cluster of points can be defined in multiple ways, we decided to use four common mathematical algorithms (convex hull, alpha shape, cubic spline, Voronoi diagram) for calculation and visual illustration of motor areas, as described in previous nTMS peer-reviewed research [18] (Figure 1).

Two assumptions were made to enable the calculation of the cortical representation areas:

1. The TMS coil stimulates the cortex area closest to the coil.
2. The TMS stimulating field is similar in every location of the cortex, and there are no relevant variations in the MEPs induced by TMS.

Thus, the 3D distribution of the stimulation locations was transformed into a 2D plane by fitting an ellipsoid to the locations. The geometric error due to the transformation concerning the distance between the individual points amounted to <1.0% and could thus be neglected.

Convex hull:

This is the smallest convex polygon containing all the locations with MEPs. However, it is limited since it does not consider negative responses within the polygon. Thus, the calculated area might appear significantly larger than its morphological representation on the cortex.

Voronoi tessellation:

So-called Voronoi cells are calculated, each representing an area of the cortex surface. Within these areas, all points are closer to the stimulated locations with positive motor answers than to locations with negative answers. Thus, the motor area is the sum of all cell areas with positive MEPs.

Alpha shape and cubic spline interpolation:

The alpha shape algorithm was implemented to better account for negative motor responses compared to convex hull and thus obtain a more realistic representation of the morphological motor cortex area. Cubic spline interpolation was added to this method to account for point clouds with comparatively large distances between the respective positive motor responses. Thus, in good correlation to the methods of Borghetti et al. and Julkunen et al., a grid with 0.1 mm spacing was generated, which contained all stimulation locations in the application of spline interpolation [18,19].

Statistical analysis was performed using the Statistical Package for Social Sciences (SPSS) 26 program (Lead Technologies, Inc., Charlotte, NC, USA).

## 3. Results

### 3.1. Patient Characteristics

We included 10 volunteers and 8 brachial plexus patients in this examination. Due to COVID-19 restrictions during the examination, we decided to limit the number of patients included. In the volunteer group, the mean age was 28 years, with 7 females and 3 males being examined. All participants were right-handed except volunteer 1 (ambidextrous) and volunteer 6 (left-handed). A detailed description of volunteer characteristics can be seen in Table 1. Here, it can be seen that we obtained a similar result for median RMT for the left and right hemispheres (37% vs. 36%). Also, the rates of positive motor responses to total stimulations applied to both hemispheres were comparable (340/2818 = 12.1% left hemisphere vs. 298/2760 = 10.8% right hemisphere).

In the patient group, the mean age was 35 years, with 2 female and 6 male patients. All patients were right-handed. One patient (patient 4) had to be subsequently excluded since no nTMS testing could be performed due to constant high muscle tone. Unfortunately, in these cases, no MEP response can be detected when a stimulus is applied, and thus, no mapping is possible. In patients with phrenic nerve transfer, mapping was quite difficult. Even 3 years after nerve transfer, we still found a breath-synchronized muscle tension pattern in EMG recordings despite a macroscopically relaxed arm. Stimulation had to be applied in between these involuntary muscle contractions to receive an adequate EMG response. However, voluntary elbow flexion was possible independent of breathing.

A detailed analysis of patient characteristics is demonstrated in Table 2.

**Figure 2:**
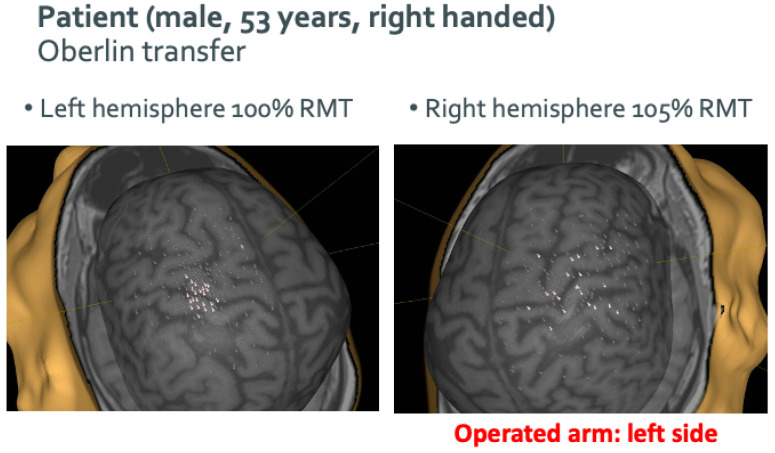
An exemplary case of nTMS mapping of a patient who received an Oberlin transfer (transferring part of the ulnar nerve onto the musculocutaneous nerve) as part of brachial plexus reconstruction surgery.

Median RMT for the hemisphere contralateral to the affected arm was 41% compared to 50% on the ipsilateral side. Both values were elevated compared to the control group (36% right, 37% left hemisphere). The rate of positive motor responses to total stimulations applied to each hemisphere was 11.8% (158/1562) on the affected hemisphere and 9.3% (146/1578) on the unaffected hemisphere. A representative example of nTMS mapping can be seen in Figure 2.

### 3.2. Cortical Motor Area Location

**Figure 3:**
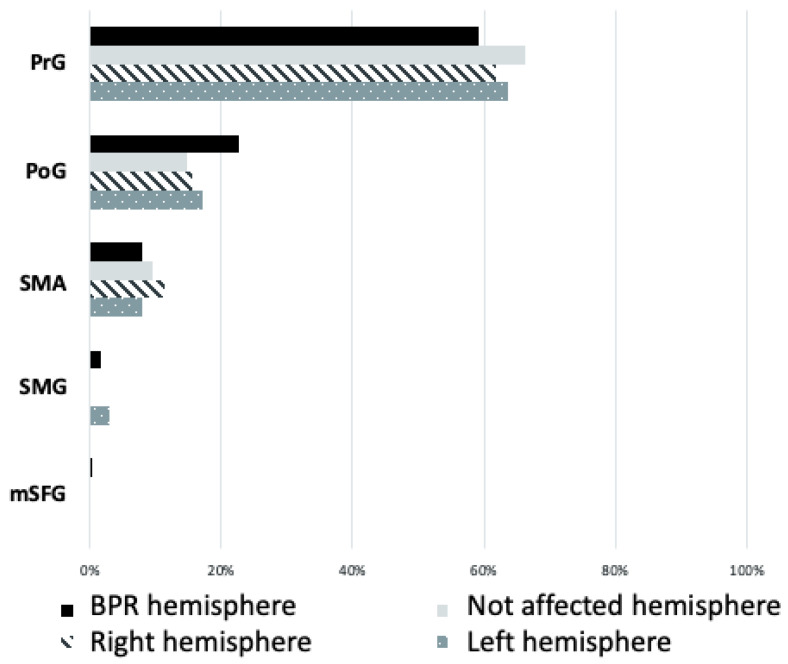
Demonstration of cortical motor distribution in brachial plexus reconstruction (BPR) patients and volunteers categorized by the gyral anatomy. Patients’ and volunteers’ responses are divided by hemisphere (“right” and “left” hemisphere for volunteers and “BPR hemisphere” and “not affected hemisphere” for patients). The cortical areas with relevant representation are the precentral gyrus (PrG), postcentral gyrus (PoG), supplementary motor area (SMA), supramarginal gyrus (SMG), and medial superior frontal gyrus (mSFG).

When analyzing where bicep motor representation iwa localized, we found that in the case of patients with brachial plexus reconstruction (BPR), 59% (±0.23) of positive motor responses could be found on the precentral gyrus (PrG). Stimulation responses on the unaffected hemisphere of patients (*n* = 7) and both hemispheres of healthy volunteers (*n* = 10) together showed a representation on PrG in 63.9% (±0.18). In the Wilcoxon test, the distribution on the precentral gyrus showed a mean of 3.3 (±4.7) for the affected hemisphere of patients and a mean of 3.0 (±5.8) for the unaffected side of patients and both hemispheres of the control subjects. These results were not significant (*p* = 0.27). An illustration of the cortical distribution of stimulation responses can be seen in Figure 3; here, distribution is categorized by the gyral anatomy.

**Figure 4:**
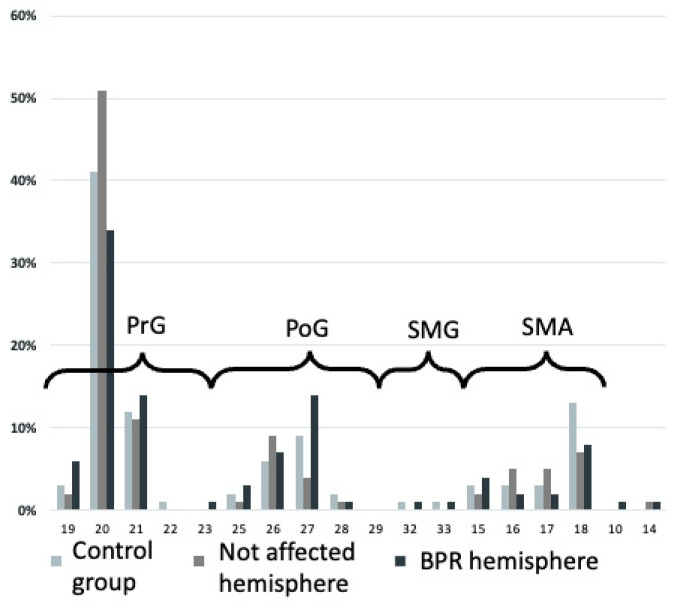
Demonstration of cortical motor distribution in brachial plexus reconstruction (BPR) patients and volunteers (control group) categorized by a cortical parcellation system proposed by Corina et al. [17]. An area with increased representation on the BPR hemisphere is, for example, number 27 (caudal medial postcentral gyrus).

Figure 4 offers a more specific look at cortical distribution using a cortical parcellation system, as described in the Methods section.

**Figure 5:**
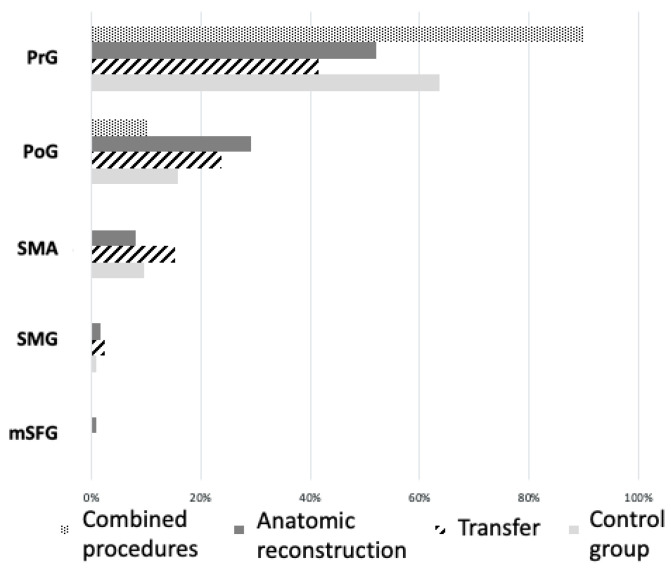
Demonstration of cortical motor distribution in brachial plexus reconstruction (BPR) patients and volunteers (control group), with patients being categorized by the type of reconstruction technique (nerve transfer, anatomical reconstruction, or a combined procedure). The cortical areas with relevant representation are the precentral gyrus (PrG), postcentral gyrus (PoG), supplementary motor area (SMA), supramarginal gyrus (SMG), and medial superior frontal gyrus (mSFG).

In both figures, the participants’ hemispheres were chosen as variables. If cortical distribution is organized by type of restoration surgery, we see a different distribution across gyri (see Figure 5).

Here, one can see that cortical representation after transfer surgery (23.8% ± 0.16 of positive motor responses) and more so after anatomical reconstruction (29.2% ± 0.11) has shifted towards the postcentral gyrus (PoG) compared to the control group (15.9% ± 0.14). In contrast, increased localization to the secondary motor areas (SMAs), defined as the posterior superior frontal gyrus (pSFG), posterior supramarginal gyrus (pSMG), and opercular part of inferior frontal gyrus (opIFG), was particularly evident with nerve transfer at 15.3% (±0.13). In comparison, only 9.6% (±0.14) of positive stimulation points were localized to this area in the control group. However, a combined surgical procedure of nerve transfer and anatomical reconstruction increased localization in the precentral gyrus with 89.8% (±0.004), which was also more pronounced than in the control group with 63.7% (±0.18).

**Figure 6:**
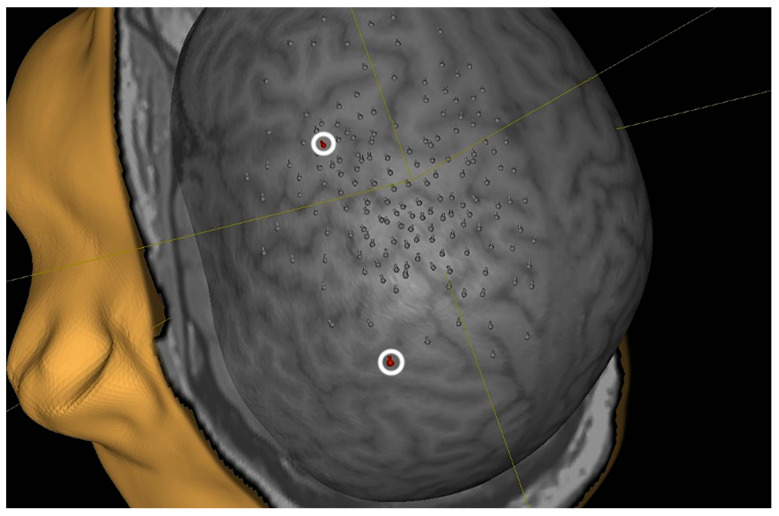
Exemplary case of sporadic ipsilateral motor activity in patients’ nTMS motor mapping, which was interpreted as two outliers (marked with white circle and red dot) after reviewing their EMG response.

As mentioned, every patient was also monitored for ipsilateral cortical bicep representation. However, apart from occasional, single outliers, we did not see ipsilateral activation clusters (Figure 6).

### 3.3. Cortical Motor Area Size

When examining the size of the cortical motor area for bicep activation, we chose to use more than one calculation model, as described in the Methods section (Figure 1). The individual results can be seen in Table A1 and Table A2. This diagram illustrates that the mean cortical bicep area of the contralesional hemisphere is increased compared to the patients’ unaffected hemisphere and the control group. This effect can be seen regardless of the chosen calculation model.

**Figure 7:**
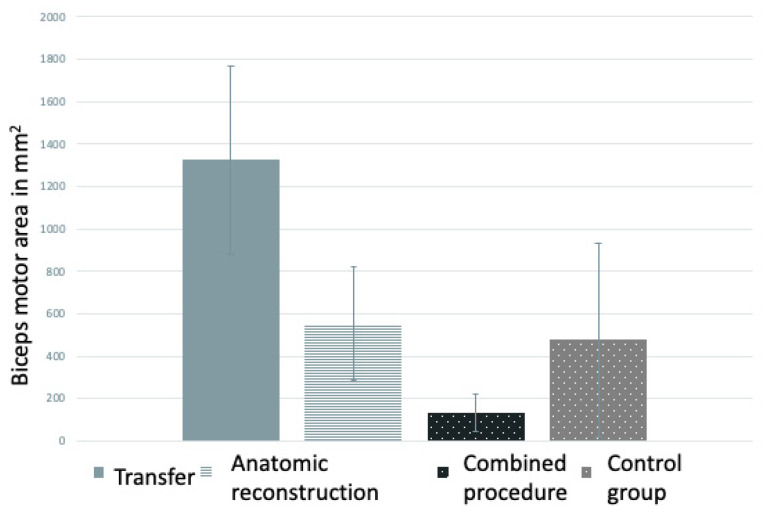
Biceps motor area size in mm^2^ grouped by type of reconstruction surgery for patients compared to healthy controls.

Similar to the cortical motor location, for cortical motor area size, organization by type of restoration surgery can be chosen, as demonstrated in Figure 7. Compared to the control group, after nerve transfer, we see an increase in size (1326 ± 443 mm^2^), whereas after anatomical reconstruction, a comparable size of the area can be seen (554 ± 266 mm^2^ for the reconstruction group and 477 ± 455 mm^2^ for the control group). We noted a decrease in cortical motor area size for patients who received a combination of both techniques (133 ± 86 mm^2^).

## 4. Discussion

In this article, we have presented a prospective study examining motor neuroplasticity changes and functional cortical reorganization after BPR surgery. While other nTMS studies so far have often examined pathologies inducing cortical neuroplasticity immediately after intervention, in our cohort, the time between deafferentation and “reafferenciation” was approximately 1 year if surgical restoration after 6 months and rapid nerve sprouting after 6 months for the musculocutaneous nerve were presumed [20]. After a “locked-in” situation of the motor network of the arm region lasting for roughly one year, neuroplastic changes might present differently than in other pathologies. In order to further understand these processes and compare them with existing knowledge, we conducted this study, producing the following main findings.

Firstly, we were able to show that nTMS mapping of patients after BPR is generally feasible, even if it can be complicated in specific cases with phrenic transfers or even impossible in patients with increased muscle tone.

Similar to other pathologies, we found that RMTs of both hemispheres showed a greater difference in the patient cohort than in the control group [21,22]. RMTs tended to be lower in the affected hemisphere compared to the contralateral side. We interpret this as a sign of increased cortical excitability of the affected hemisphere. However, in general, patients had a higher RMT level than our control group. This could be attributed to the high rate of associated traumatic brain injuries in our patient cohort, as brachial plexus lesions are often caused during high-velocity traffic accidents. This phenomenon has been described in the previous nTMS literature [23].

Jussen et al. and Acker et al. showed that patients with vascular pathologies (occlusive cerebrovascular disease and moyamoya) preoperatively present had an increased RMT, which readapted to the contralateral hemisphere after revascularization [24,25]. In our case, no preoperative status could be evaluated since, in a plegic extremity, no MEPs can be elicited, and therefore, only postoperative mapping is possible. Examinations with available pre-lesional nTMS data are very rare [26].

Zdunczyk et al. examined cortical excitability and reorganization in patients with different severity levels of cervical myelopathy [27]. Concerning RMT, they did not see a difference between patients and their control group.

In stroke, some trials report an unchanged RMT compared to the unaffected hemisphere [28], and others describe a realignment after initial asymmetry with increased RMT of the affected hemisphere [22].

Furthermore, confirming our hypothesis, motor area location after surgical reconstruction could primarily be found at the precentral gyrus. A shift of motor representation, even if not statistically significant, could be seen towards the SMA region for nerve transfers and towards the postcentral gyrus for anatomical reconstructions.

A neuroplastic shift of motor function towards the premotor cortex has been observed in different pathologies like stroke or tumors. Fridman et al. report a reorganization of motor function at the dorsal premotor cortex after stroke in the primary motor cortex [9]. The same phenomena can be examined in brain tumor patients, as Bulbas et al. report [29].

Forster et al. also demonstrated a postoperative shift along the anterior–posterior axis after low-grade glioma surgery, confirmed by Conway et al., who have shown that a shift in the dorsal direction is also possible [10,30].

In sum, the reorganization at the precentral gyrus and partial shift of motor function towards secondary motor areas seems to be in line with prior research and may be well-explained by the neuroplastic changes necessary, especially after nerve transfer procedures.

Lastly, we found an increase in cortical motor area size in the affected hemisphere compared to the contralateral and control group hemispheres, especially in patients with nerve transfers. In our opinion, a larger cortical area for nerve transfers is well-explained by increased cortical “recruitment” compared to a mere “restoration of nerve continuity” in anatomical reconstruction, where the cortical area is similar to the control group. A decrease in size in combined procedures (for example, C5 and pectoral nerve on the musculocutaneous nerve), however, is more surprising. Maybe the possibility of two “pathways” (C5 root and pectoral nerve) requires less cortical area. However, a definite statement would require a much bigger sample size than our cohort.

Looking at the literature and findings in other pathologies, Acker et al. describe an initially enlarged cortical representation in moyamoya patients, which decreases after revascularization, and similarly, Jussen et al. report a decrease in cortical representation area of occlusive cerebrovascular disease patients after revascularization [24,25]. The same research group has also examined motor area changes in case of cervical myelopathy and, in this regard, firstly proposed the term “corticospinal reserve capacity”, where an enlargement of motor area size and decrease in RMT can be seen until a state of decompensation is reached where both attributes reverse until no MEP can be elicited [27]. Following this theory, our findings could be interpreted as a reversal of the mentioned path, ending in a recompensated state (large motor area, low RMT) comparable with beginning cervical myelopathy or untreated moyamoya.

Further studies, like nTMS motor mapping of brachial plexus patients at multiple points in time during their recovery, are needed to further clarify this theory. Studies by Mano et al. and Chen et al. also show some resemblance to our examination [31,32]. Mano et al. examine central motor reorganization after anastomosis of the musculocutaneous and intercostal nerves following cervical root avulsion in a cohort of four patients. The researchers describe mapping at three to four time points during recovery. However, only non-navigated location data are provided, and no motor area sizes are examined. The team find a decrease in MEP latencies and a more lateral relocation of the motor area over time.

Chen et al. analyze motor plasticity after gracilis muscle transfers in nine cases. The time of examination varies from 4 months to 23 years after surgery. Motor information is gathered with conventional TMS and functional MRI (fMRI). From this, Chen et al. find a reduction in RMT, interpreted as a sign of disinhibition. However, they do not see an increase in motor area or change in motor area location compared to the non-operated side. To frame how these findings should be interpreted, we note that Chen’s examination lacks a control group and that nTMS offers more accuracy than TMS.

Lastly, the presence of breathing-synchronized EMG activity after phrenic nerve transfer should be discussed. It is a phenomenon described in the literature for phrenic nerve transfers [33]. In its extreme form, the “breathing arm” can be witnessed with not only EMG activity but also visible muscle contractions. Socolovsky eloquently describes the historical development of the term [34]. The question of the physiological reason, however, invites speculation. In our opinion, this phenomenon serves as evidence for the neuroplastic process that has taken place. On the one hand, prominent MEP activation can now be achieved via the motor cortex, the new home of bicep representation. On the other hand, the phrenic nerve has never forgotten its origin in the autonomic respiratory center. Thus, motor function may exist in two places simultaneously, maybe documenting only a partial migration of function.

### 4.1. Limitations

In this article, we could only present data from a small number of patients since we found that patient recruitment without remuneration during the COVID-19 pandemic was quite challenging. This small sample was also imbalanced—unlike our patient group, our control group consisted of more females than males. Additionally, in retrospect, further parameters like the vicinity of stimulation responses (e.g., objectified by a virtual grid) would have been interesting to collect. Finally, we unfortunately could only present one mapping per patient. Demonstrating dynamic neuroplastic changes over time would have given further insight. 

### 4.2. Future Perspectives

Understanding cortical reorganization of the motor area after brachial plexus surgeries is important for optimal postoperative support. In order to further improve treatment, neuroplasticity-inducing measures seem to be a logical next step. Further studies should examine whether, for example, therapeutic postoperative nTMS stimulation may be a viable option. Furthermore, a deeper understanding of cortical motor organization can serve as basis for brain–machine interfaces (BMIs) in the future. Mechanical hand orthoses and exoskeletons are already being used in brachial plexus cases where a surgical solution was unfortunately not possible, and improving these devices with cortical motor data could represent a significant enhancement.

## 5. Conclusions

In this study, we were able to demonstrate that nTMS motor mapping after BPR is feasible and shows a relocation of bicep brachii representation at the middle precentral gyrus of the corresponding contralateral hemisphere. Motor area size is increased compared to the control group and the patient’s unaffected, ipsilateral hemisphere. RMTs were lower in the affected hemisphere than in the unaffected hemisphere. The issue of motor neuroplasticity in patients with brachial plexus lesions is worth exploring in further studies.

## Figures and Tables

**Table 1:** Volunteers’ characteristics.

Volunteer	Sex	Age	Handedness	RMT, Left	Total Number of Stimulations, Left	Positive Motor Responses, Left	RMT, Right	Total Number of Stimulations, Right	Positive Motor Responses, Right
volunteer 1	f	29	ambidextrous	33%	273	33	37%	226	33
volunteer 2	f	41	right	42%	173	32	32%	240	9
volunteer 3	m	31	right	36%	373	14	35%	398	19
volunteer 4	f	23	right	31%	326	23	34%	327	15
volunteer 5	m	25	right	68%	295	73	51%	339	34
volunteer 6	f	24	left	29%	297	31	32%	258	30
volunteer 7	f	33	right	34%	368	23	32%	331	22
volunteer 8	m	24	right	38%	258	27	60%	175	54
volunteer 9	f	25	right	38%	199	14	45%	205	38
volunteer 10	f	24	right	49%	256	70	48%	261	44
sum					2818	340		2760	298
mean		27.9		40%			41%		
median				37%			36%		

**Table 2:** Patient data.

Patient	Sex	Age	Age at Accident	Handedness	Affected Hemisphere	Associated TBI	Detailed Procedure	Type of Reconstruction	RMT of Affected Hemisphere	Total Number of Stimuli on Affected Hemisphere	Positive Motor Responses on Affected Hemisphere	RMT of Unaffected Hemisphere	Total Number of Stimuli on Unaffected Hemisphere	Positive Motor Responses on Unaffected Hemisphere
patient 1	m	52	45	right	right	yes	Oberlin transfer	transfer	37%	184	20	59%	172	25
patient 2	m	29	25	right	right	yes	phrenic nerve on biceps branch of MC + C5 on brachial branch of MC	combination	36%	184	19	49%	256	18
patient 3	f	41	32	right	left	yes	phrenic nerve on MC	transfer	84%	77	32	99%	74	8
patient 4	m	27	17	right	left	yes	neurolysis	neurolysis						
patient 5	f	38	31	right	left	no	C5 on MC + pectoral nerve on MC	combination	44%	229	10	48%	212	31
patient 6	m	33	25	right	left	no	lateral fascicle on MC	anatomical reconstruction	37%	269	42	35%	225	12
patient 7	m	25	18	right	left	no	C6 on MC	anatomical reconstruction	32%	313	41	39%	285	23
patient 8	m	36	26	right	left	yes	lateral fascicle on MC	anatomical reconstruction	60%	306	21	51%	354	29
sum										1562	185		1578	146
mean		35.1							47%			54%		
median									41%			50%		

## Data Availability

Data available on demand.

## Appendix A

**Table A1:** The first part of our detailed calculations of the motor area size.

		3D Convex Hull		2D Convex Hull		2D Voronoi		2D Alpha	
Category	ID	Left	Right	Left	Right	Left	Right	Left	Right
volunteer	**1**	197	362	192.9	339.8	144.7	350.4	123.5	279.1
volunteer	**2**	682.6	45	644.6	36.9	510.3	26.1	571.8	27.7
volunteer	**3**	243	132	239.1	119.5491	70.3	82.6644	170.1	88.5177
volunteer	**4**	227	162	220.2	110.3	106.9	79.7	179.7	99.4
volunteer	**5**	1643	361	1568.5	332.4	974	193.3	1260.5	279.4
volunteer	**6**	299	433	273.4	369.9	186.2	289.9	203.8	316
volunteer	**7**	349	378	337.2	350.4	139.3	132.8	296.6	181.9
volunteer	**8**	380	1376	370.5	1083.5	174.1	1284.6	239.2	934.5
volunteer	**9**	183	415	181.8	402.3	97.7	308	100	300.9
volunteer	**10**	526	384	502.3	358.9	425.1	225.6	380.9	283.7
mean		473.0	404.8	453.1	350.4	282.9	297.3	352.6	279.1
**Category**	**ID**	**unaffected**	**affected**	**unaffected**	**affected**	**unaffected**	**affected**	**unaffected**	**affected**
patient	**1**	536.9	1639	525.7	1491.7	270	399.2	436.2	1105.3
patient	**2**	1157.3	194	1132.2	190.5	278.9	86	952.8	167.5
patient	**3**	177	1013	125	953.5	177.2	1252.2	103.6	686
patient	**5**	290	72	286.4	63.2	174.8	65.5	260.2	45.6
patient	**6**	344.8	449.8	236.5	435.2	119.1	394.7	195.9	381.5
patient	**7**	359.8	735.2	340.4	620.4	156.6	459.3	315.6	510.7
patient	**8**	143	451	134.6	441.8	103.8	126.7	123.4	352
mean		429.8	650.6	397.3	599.5	182.9	397.7	341.1	464.1

**Table A2:** The second part of our detailed calculations of the motor area size.

		2D Interpol		SD ABV		SD BLW		SD Tot	
Category	ID	Left	Right	Left	Right	Left	Right	Left	Right
volunteer	**1**	123.3	276.6	−0.53	−3.94	−1.14	−2.41	−1.44	−2.55
volunteer	**2**	640.6	38.1	−2.12	−0.70	−2.26	−2.89	−2.24	−2.91
volunteer	**3**	200.5	94.284	−0.81	−5.29	−2.19	−2.84	−2.22	−2.90
volunteer	**4**	199.9	104	−1.52	−1.30	−2.01	−2.82	−2.05	−2.88
volunteer	**5**	1276.8	283.3	−1.17	−3.54	−1.60	−2.14	−1.62	−2.17
volunteer	**6**	206.9	332.7	−1.46	−7.29	−2.27	−2.50	−2.37	−2.60
volunteer	**7**	307.5	196.6	−0.65	−2.08	−2.11	−2.75	−2.16	−2.79
volunteer	**8**	252.4	933.4	−0.20	−1.66	−1.47	1.17	−1.51	−2.10
volunteer	**9**	98.7	312	−0.21	−1.51	−1.71	−2.89	−1.71	−3.03
volunteer	**10**	372.3	279.4	−1.34	−0.69	−2.61	−2.93	−2.89	−3.11
mean		367.9	285.0	−1.00	−2.80	−1.94	−2.30	−2.02	−2.70
**Category**	**ID**	**unaffected**	**affected**	**unaffected**	**affected**	**unaffected**	**affected**	**unaffected**	**affected**
patient	**1**	470.7	1274.2	−0.37	−1.38	−1.14	−3.06	−1.67	−3.07
patient	**2**	1109.9	194	−0.39	−0.48	−1.28	−2.28	−1.28	−2.35
patient	**3**	102.7	695	−2.17	−1.66	−3.09	−1.40	−3.13	−1.44
patient	**5**	277.8	50.8	−0.29	−1.38	−2.38	−2.31	−2.46	−2.36
patient	**6**	232.3	385	−0.49	−0.98	−1.60	−1.50	−1.62	−1.58
patient	**7**	324.6	527.1	−1.32	−1.19	−1.08	−1.73	−1.09	−1.79
patient	**8**	117.7	363.1	−1.54	−0.49	−3.13	−2.78	−3.18	−2.80
mean		376.5	498.5	−0.94	−1.08	−1.96	−2.15	−2.06	−2.20
